# High Glucose Induced Alteration of SIRTs in Endothelial Cells Causes Rapid Aging in a p300 and FOXO Regulated Pathway

**DOI:** 10.1371/journal.pone.0054514

**Published:** 2013-01-16

**Authors:** Rokhsana Mortuza, Shali Chen, Biao Feng, Subhrojit Sen, Subrata Chakrabarti

**Affiliations:** Department of Pathology, Schulich School of Medicine & Dentistry, University of Western Ontario, London, Ontario, Canada; University Hospital Freiburg, Germany

## Abstract

In diabetes, some of the cellular changes are similar to aging. We hypothesized that hyperglycemia accelerates aging-like changes in the endothelial cells (ECs) and tissues leading to structural and functional damage. We investigated glucose-induced aging in 3 types of ECs using senescence associated β-gal (SA β-gal) staining and cell morphology. Alterations of sirtuins (SIRTs) and their downstream mediator FOXO and oxidative stress were investigated. Relationship of such alteration with histone acetylase (HAT) p300 was examined. Similar examinations were performed in tissues of diabetic animals. ECs in high glucose (HG) showed evidence of early senescence as demonstrated by increased SA β-gal positivity and reduced replicative capacities. These alterations were pronounced in microvascular ECs. They developed an irregular and hypertrophic phenotype. Such changes were associated with decreased SIRT (1–7) mRNA expressions. We also found that p300 and SIRT1 regulate each other in such process, as silencing one led to increase of the others’ expression. Furthermore, HG caused reduction in FOXO1’s DNA binding ability and antioxidant target gene expressions. Chemically induced increased SIRT1 activity and p300 knockdown corrected these abnormalities slowing aging-like changes. Diabetic animals showed increased cellular senescence in renal glomerulus and retinal blood vessels along with reduced SIRT1 mRNA expressions in these tissues. Data from this study demonstrated that hyperglycemia accelerates aging-like process in the vascular ECs and such process is mediated via downregulation of SIRT1, causing reduction of mitochondrial antioxidant enzyme in a p300 and FOXO1 mediated pathway.

## Introduction

Diabetes and its complications account for significant morbidity and mortality throughout the world [1]–[3]. The major factor in the development of chronic diabetic complications is vascular EC dysfunction [4]. The prevailing mechanism leading to EC dysfunction is an increase in reactive oxygen species (ROS) formation [5]. In response to high ambient glucose levels and subsequent oxidative stress, ECs elaborate large amount of vasoactive factors, growth factors and cytokines [6], [7]. Such factors lead to increased production of extracellular matrix (ECM) proteins causing structural alterations [6]–[8]. Interestingly, several such changes seen at the cellular and tissue level in diabetes are similar to the changes seen in normal aging process [9]–[13].

Oxidative stress causes DNA damage and alters transcriptional machinery both in aging and in diabetes [4], [6], [14], [15]. We have previously shown that glucose induced oxidative stress causes histone acetylation by p300, which regulates several transcripts in diabetes [6], [16]. p300, a transcriptional coactivator with an intrinsic histone acetyltransferase (HAT) activity, regulates numerous transcription factors [6], [16], [17]. Acetylation by p300 and other HATs are balanced by histone deacetylases (HDACs).

Silent information regulator 2 proteins or sirtuins (SIRTs) belong to Class III HDACs and regulates epigenetic gene silencing and suppress recombination of rDNA [18]–[20]. In mammals, SIRTs have a range of molecular functions and have emerged as important proteins in aging and metabolic regulations [18], [21]. SIRTs represent a small gene family with seven members designated as SIRT1–7, known to be modulated by oxidative stress [22].

Some of the SIRTs activity is carried out through deacetylation of the FOXOs, forkhead family ‘O’ group of transcription factors [23]–[25]. Among the FOXO family, FOXO1 is best characterized and plays important roles in cell survival, oxidative stress resistance and cell death [26]–[29]. FOXO1 has a highly conserved DNA binding domain subjected to posttranslational modifications such as phosphorylation, acetylation and ubiquitination. These modifications can either increase or decrease the transcriptional activity of FOXO1 [17]. FOXO1 acetylation by HAT such as p300, leads to attenuation of its DNA binding ability and facilitates its phosphorylation by Akt, leading to its export from the nucleus; whereas deacetylation increases FOXO1’s transcriptional activity [17], [24].

The purpose of this study was to investigate whether high glucose causes accelerated aging process in ECs through alteration of SIRTs. We further investigated whether the effects of SIRTs are mediated through FOXO1 and if such process is regulated by histone acetylase p300. We carried out these studies in various ECs as well as in the diabetic animals.

## Methods

### Cell Culture

Dermal-derived human microvascular EC (HMEC) was obtained from Lonza, Inc. (Walkersville, MD) and grown in EC basal medium 2 (EBM-2, complete). Human umbilical vein ECs (HUVECs) were obtained from Lonza and cultured in EC growth medium (EBM complete, Walkersville, MD). Bovine retinal microvascular ECs (BRECs) were obtained from VEC Technologies (Rensselaer, NY) and grown in a defined EC growth medium (MCDB-131 complete). We have previously described the culture conditions of these three cells [30], [31]. No insulin was present in any media.

For the long term continuous exposure to glucose, ECs were cultured in 12 well plates (Corning, Acton, MA) and treated with 5 mM glucose (NG) or 25 mM glucose (HG, D-glucose) or osmotic control (OSM, 25 mM L-glucose). Upon confluence cells were propagated & maintained in the same treatment condition until they stopped proliferating completely. During each passage subculture cells from each treatment group were stained for SA β-gal and collected for RNA analysis. Cell lysates were collected with RIPA (Millipore, Billerica, MA) buffer with protease inhibitor (Roche, Laval, Canada) for ROS and MnSOD analysis. Total protein concentrations were measured by BCA protein assay kit (Pierce, Rockford, IL). Cells were monitored daily and images taken for morpholocigal and growth analysis. All experiments were conducted with 6–10 biological replicates.

To test the effect of SIRT1 activation on accelerated aging in diabetes, cells were treated with 10 µM resveratrol (Sigma, Oakville, ON, Canada) dissolved in ethanol or 25 µM BML278 (Enzo, Farmingdale, NY) in DMSO for 72 hr in HG following subculture in HMEC P1 (P1 =  passage 1). To investigate the effect of FOXO1 inhibition, cells were treated with 0.1 µM FOXO1 inhibitor AS1842856 (Millipore, Billerica, MA) in DMSO similarly.

### Animal Experiments

Male C57BL/6 mice (20–25 g), were obtained (Charles River, Wilmington, MA) and diabetes was induced by a single intraperitoneal injection of streptozotocin (STZ) (65 mg/kg, in citrate buffer, pH 5.6). Age- and sex-matched mice were used as controls and given equal volumes of citrate buffer [32]. *db/db* (Lepr^db^, DBA/J) mice (8 weeks, Jackson Laboratory, CA) were used as type 2 model of Diabetes [32]. The animals were monitored daily as described by us previously [32]. The animals were killed at 2 & 4 months following the development of diabetes (n = 10/group). Retinal and renal cortical tissues were dissected out and snap frozen in liquid nitrogen. All tissues were stored at −80°C until further analysis. Urinary albumin (Exocell, Philadelphia, PA) and serum creatinine (Arbor assays, Ann Arbor, MI) were measured as per the instructions.

### Ethics Statement

Animal experiments were performed in accordance with regulations specified by the Canadian Council of Animal Care. The investigation was in compliance with the Guide for the Care and Use of Laboratory Animals (NIH publ. no. 85–23, revised 1996). All protocols were approved by the University of Western Ontario Animal Care and Veterinary Service.

### SA β-gal Staining of Cells and Frozen Tissue Sections

Cells from each passage or tissue slides were fixed and stained with SA β-gal staining according to the manufacturer’s instructions (abcam, Cambridge, MA). Tissue slides were counterstained with H&E for orientation purpose.

### Phase Contrast Microscopy and Morphometrical Analysis

Images of SA β-gal stained cells were photographed with phase contrast inverted microscope (Meiji Techno, TC5400, Santa Clara, CA) with 20× objective and SPOT Basic software. Morphometrical analysis of the images was done by ImageJ software (NIH, Bethesda, MD). Images (10 per sample) of the tissue slides were recorded by an Olympus BX51 microscope (Olympus, Center Valley, PA) with Northern Eclipse software (Empix Inc, Cheektowaga, NY).

### mRNA Extraction and cDNA Synthesis

RNA extraction and cDNA synthesis from cells and tissues samples has been described by us previously [30]. RNA concentration was assessed on a spectrophotometer (Pharmacia Gene Quant, GE, Mississauga, ON, Canada). First-strand cDNA was made by using High Capacity cDNA Reverse Transcription kit (Applied Biosystems, Carlsbad, CA, USA) as per the manufacturer instruction.

### mRNA Analysis with Quantitative Real-time RT-PCR

Real-time RT-PCR was performed by LightCycler™ (Roche Diagnostics, Laval, Canada) to quantify the mRNA expression of SIRT1, SIRT2, SIRT3, SIRT4, SIRT5, SIRT6, SIRT7, TERT (telomerase reverse transcriptase) and p300 using the Qiagen One Step RT-PCR kit (SYBR Green I detection platform). All primers were either ordered or custom made from Sigma (Table S1). The data were normalized to housekeeping gene β-actin/18s mRNA to account for differences in reverse transcription efficiencies and the amount of template in the reaction mixtures. The details of the method have been described by us previously [30].

### Nuclear Fraction Isolation

Nuclear fractions were isolated from ECs and tissues as per the kit instruction (Active Motif, Carlsbad, CA). Protein concentrations in the samples were measured by BCA protein assay (Pierce, Rockford, IL).

### FOXO1 DNA Binding Activity Measurement

FOXO1 DNA binding ELISA was conducted on the collected nuclear fraction as per the manufacturer’s instruction (Active Motif, Carlsbad, CA). The plates were read at 450 nm using a plate reader (Multiskan, Thermo Fisher, Canada).

### ROS, MnSOD and LDH Analysis

Total ROS level in the EC lysates were measured as per the manufacturer instructions using a commercially available kit (Cell Biolabs Inc., San Diego, CA). The plates were read with a fluorescent plate reader (Biotek, Winooski, VT) at excitation 480 nm and emission 530 nm. MnSOD ELISA was done on cell and tissue lysates as per the manufacturer instruction (abcam, Cambridge, MA). The plates were read at 450 nm using a plate reader (Multiskan, Thermo Fisher, Canada). Total protein concentrations were measured by BCA protein assay kit (Pierce, Rockford, IL). Quantification of intracellular lactate dehydrogenase (LDH) in the cell lysates was used as an additional measure of cell growth. The assay was conducted as per the manufacturer instruction (Caymen Chemical Company, Ann Arbor, MI).

### Telomerase Activity

Telomerase activity of the samples were measured using a commercially available kit following instructions provided by the manufacturer (Allied Biotech Inc., Vallejo, CA).

### Western Blot

200 µg of protein was used for the western blot analysis according to the standard protocol established at our lab [6] using p300, Ac-FOXO1 & β-actin antibody (Santa Cruz Biotechnology, Santa Cruz, CA).

### SIRT1 Enzyme Activity Analysis

Enzyme assay for SIRT1 activity was performed as per the manufacturer instructions (Sigma, Oakville, CA). The plates were read with a fluorescent spectrophotometer (Biotek, Winooski, VT) at excitation 340 nm and emission 430 nm.

### SIRT1 Gene Silencing

To silence SIRT1, transfection of small interfering RNA (siRNA) was performed using N-TER nanoparticle siRNA transfection system (Sigma, Oakville, Canada) according to the manufacture’s protocol. SIRT1 siRNA1 was purchased from Dharmacon Inc. (Lafayette, CO) and SIRT1 siRNA2 was purchased from Santa Cruz Biotecnology (Santa Cruz, CA). Cells were incubated with or without glucose after transfection for 24 hr and sample collected for mRNA analysis. To test the effect of SIRT1 siRNA on resveratrol, the siRNA was added 48 hr post treatment with resveratrol in the cultured cells and samples collected 24 hr post transfection. Transfection efficiency was assessed by real-time RT-PCR.

### p300 Gene Silencing

To silence the p300 expression in ECs, p300 siRNA1 (Silencer, Ambion, Austin, TX) and p300 siRNA2 (Santa Cruz Biotechnology, Santa Cruz, CA) was used with siPORT Lipid transfection reagent (Ambion, Carlbad, CA). The details of the transfection protocol has been described by us previously [6], [16]. siRNA transfection efficiency was indirectly assessed by measuring p300 mRNA expression by real-time RT-PCR.

### Statistical Analysis

Data are expressed as mean ± SEM (n≥6), normalized to controls. The statistical significance of the results was analyzed by one way or two way ANOVA followed by Tukey’s HSD post hoc correction and the two tailed Student’s t-test as appropriate (PASW Statistics 18, IBM, Canada). P value <0.05 was considered statistically significant.

## Results

### High Glucose Accelerates Aging Process in the ECs

Suspecting that hyperglycemia accelerates aging, we first investigated whether glucose causes rapid aging process in the ECs as they are the primary target of diabetic vascular complications. We examined three different ECs of various origins, at various passages following treatment with high glucose and used morphological alteration & senescence associated β-gal positivity as two biomarkers of aging.

**Figure 1:**
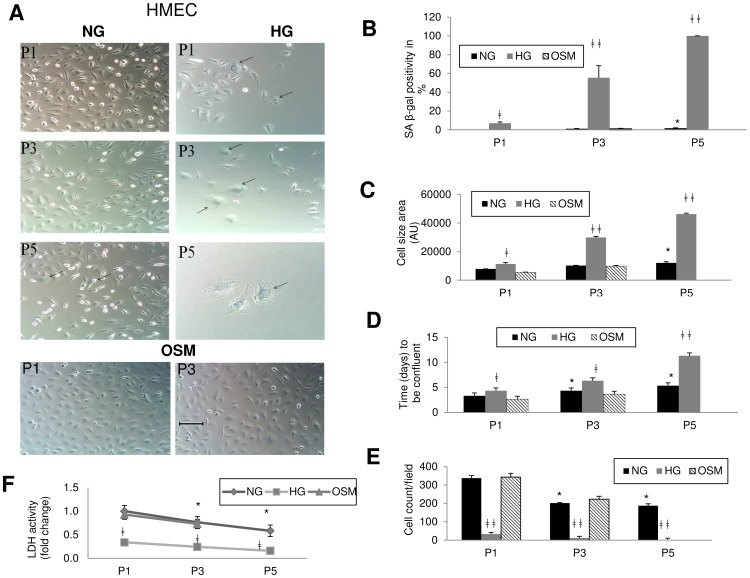
Aging signs in microvascular endothelial cells (HMEC) with HG starts as early as passage 1. (A) SA-βgal staining showed increased β-gal positivity or senescent cells with HG treatment compared to the NG treated cells, with the increasing passage number. Arrow indicates β-gal positive (blue) cells. [Scale bar represent 100 µm for all micrographs]. (B) Quantification of β-gal positivity (n = 10 image per sample). Signs of cellular aging started to appear from passage 1 in HG. (C) Morphometric cell area analysis showed significant increase in cell size with HG treatment. Cell growth was slow with HG treatment compared to controls as seen from (D) days needed to be confluent in HG (90%, at which stage cells were subcultured to next passage), (E) cell count in HG (attached cells per microscopic field at 20× objective, n = 10 image) and (F) reduced intracellular LDH activity in HG. (NG = normal glucose, 5 mM; HG = high glucose, 25 mM D-glucose; OSM = osmotic control, 25 Mm L-glucose; P = passage number). [*p<0.05 compared to NGP1; ‡p<0.05, ‡‡p<0.01 compared to respective NG passages for HG cultured cells].

We found aging changes in HMEC starts as early as passage 1, as seen from positive β-gal staining in HG treated cells compared to NG treated cells where it appeared in passage 4. By passage 5, all cells in HG showed such changes, whereas in NG it was present in only 1.5% cells (Figure 1A and B). Additionally, cell growth was slower in HG treated groups compared to the NG treated cells. By passage 5, the HG treated cells stopped proliferating completely, whereas the cells in NG continued to do so (Figure 1D–F). The morphology of the cells also changed as they got older. The older cells became larger and irregular in shape compared to the smaller spindle shaped young cells. This process was exaggerated in HG. The average cell size in HG was significantly larger than the controls (Figure 1C).

We then examined retinal endothelial cells, a major target in diabetic retinopathy. We found similar changes. In HG, BREC showed increased β-gal positivity and slower growth (Figure S1A and D–F). Similar to HMEC, such aging started as early as passage 1 in HG treated cells whereas with NG it appeared at passage 4. At passage 8 complete growth arrest and 100% β-gal positivity was seen in HG compared to 35% positivity in NG treated cells (Figure S1B). Furthermore, morphologically such aging changes were associated with a large and irregular appearance (Figure S1C).

**Figure 2:**
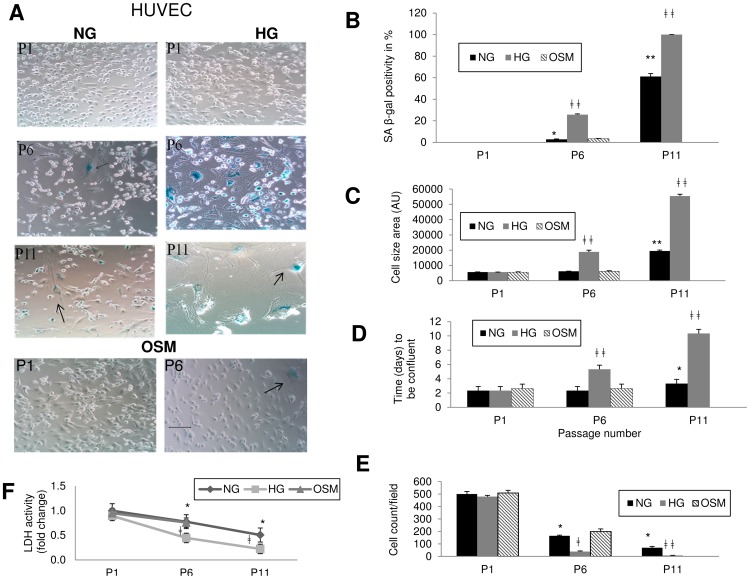
Accelerated aging in large vessel endothelial cells (HUVEC) with HG is delayed. (A) SA-βgal staining showed increased β-gal positivity (blue cells as indicated by arrow) with HG treatment compared to NG and osmotic control groups. Aging signs appeared later with HG in HUVEC. [Scale bar represent 100 µm for all micrographs]. (B) Quantification of β-gal positivity shows increased percentage of aged cells in HG treated group. (C) Morphometric cell area analysis showed significant increase in cell size with HG treatment. Cell growth was slow with HG treatment compared to controls as seen from (D) days needed to be confluent in HG (90%, at which stage cells were subcultured to next passage), (E) cell count in HG (attached cells per microscopic field at 20× objective, n = 10 image) and reduced (F) intracellular LDH activity in HG. (NG = normal glucose, 5 mM; HG = high glucose, 25 mM D-glucose; OSM = osmotic control, 25 mM L-glucose; P = passage number). [*p<0.05, **p<0.01 compared to NGP1; ‡p<0.05, ‡‡p<0.01 compared to respective NG passages for HG cultured cells].

Interestingly, although HUVECs showed similar changes, such process was delayed. HUVECs were a better survivor in HG environment. They were able to grow up to 11 passages in HG (Figure 2A). They showed signs of aging with HG at passage 4 and with NG at passage 7. β-gal positivity reached 100% at passage 11 in HG compared to 60% in NG at the same passage (Figure 2B). In addition, similar to other ECs, aging was associated with large and irregular shape of these cells along with slow growth, (Figure 2C and D–F). Such changes were not seen with 25 mM L-glucose (osmotic control) in any of the cell types (Figure 1, Figure S1 and Figure 2).

**Figure 3:**
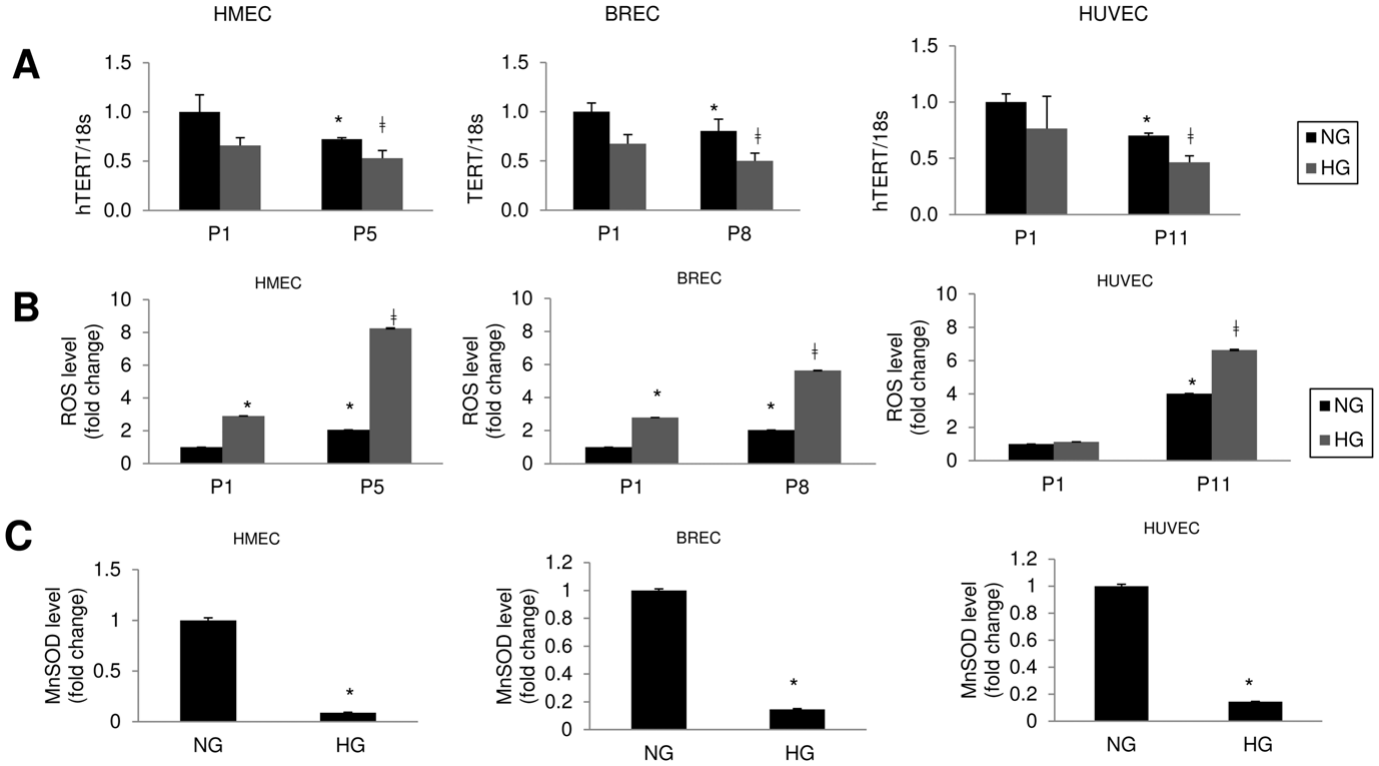
Increased oxidative stress and reduced TERT mRNA expression observed in endothelial cells with HG. (A) HMEC, BREC & HUVEC showed reduction of TERT (telomerase reverse transcriptase) mRNA in HG treated cells. mRNA levels are expressed as a ratio to 18s and normalized to NG P1. (B) Total ROS levels in HMEC, BREC & HUVEC showed significant increase with HG and aging. MnSOD levels in all cells at end passages (P5 in HMEC, P8 in BREC and P11 in HUVEC) showed significant reduction with HG treatment (C). (NG = 5 mM; HG = 25 mM glucose, P1 =  passage 1). Data normalized to NG (P1) treatments of the respective ECs. [*p<0.05 compared to NGP1; ‡p<0.05 compared to NGP5 for HMEC, NGP8 for BREC and NGP11 for HUVEC].

As reduction of TERT has been found in many models of cellular aging, we investigated possible alteration in TERT mRNA expression. We investigated them at an early stage, (P1) and at the end passage (P5 in HMEC, P8 in BREC and P11 in HUVEC). We noticed TERT mRNA was significantly reduced in all ECs with HG treatment along with accelerated aging (Figure 3A).

In order to see whether such accelerated aging is associated with oxidative stress, we measured total ROS level in the cell lysate from early and late passages between the treatment groups. As anticipated, we found with HG the ECs produced more ROS than with NG, depicting higher level of oxidative stress in hyperglycaemia, which augmented with the increased passage number or aging (Figure 3B).

Disruption of the scavenging of ROS in the mitochondria in aging is well known [33]. Manganese superoxide dismutase (MnSOD) is the primary mitochondrial ROS scavenging enzyme. It converts superoxide to hydrogen peroxide which is eventually converted to water by catalases and other peroxidases [29], [33], [34]. Our investigation of MnSOD in the final passages of the ECs showed a significant reduction of the enzyme in the aged cells (Figure 3C).

### High Glucose Exaggerates Down Regulation of SIRTs in Aging

**Figure 4:**
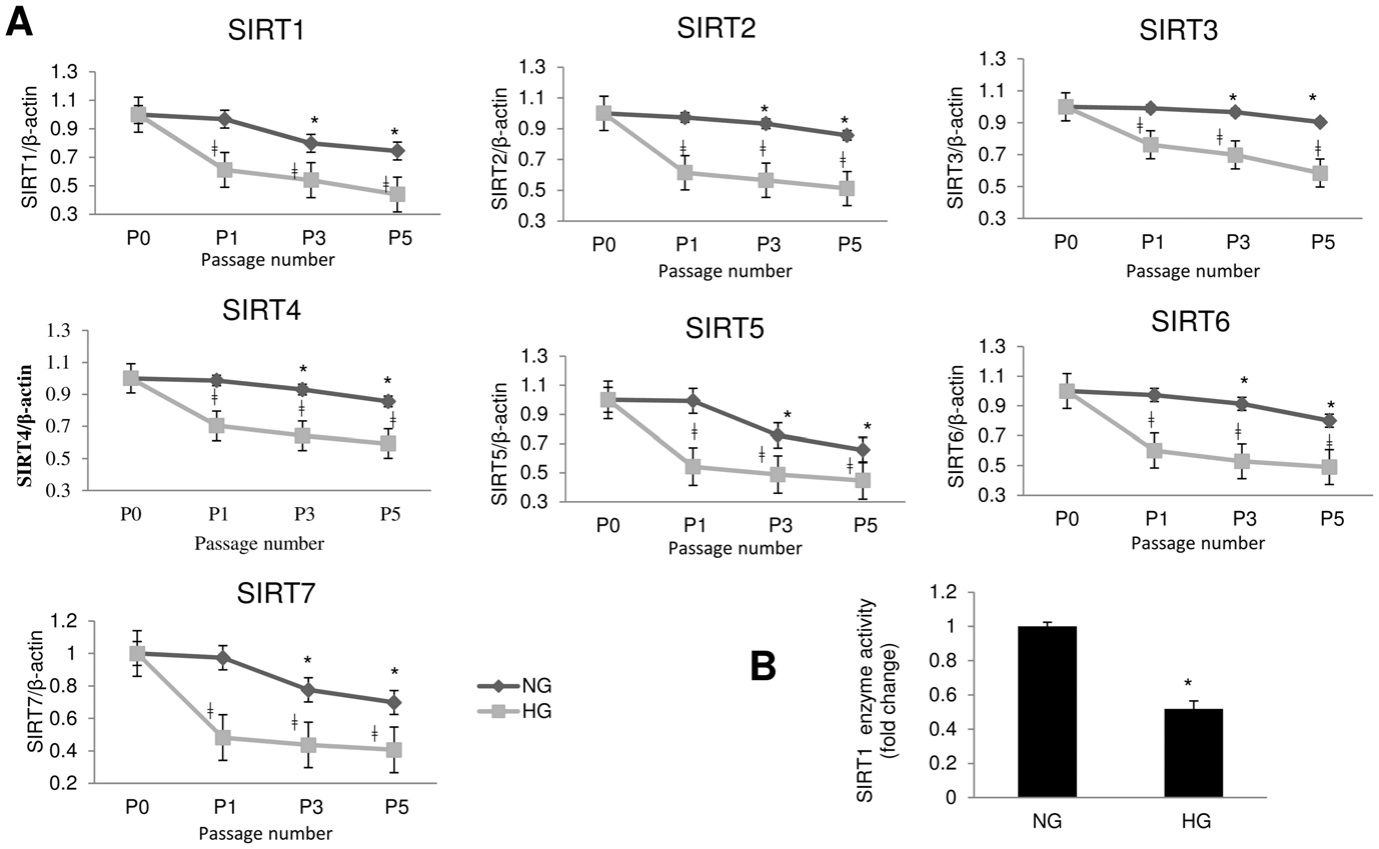
SIRT (1–7) mRNA reduction in HG in microvascular endothelial cells (HMECs) parallels the accelerated aging. (A) Quantitative Real Time RT-PCR of SIRTs in HMEC showed significant reduction in the SIRT mRNA levels in HG treated cells. mRNA levels are expressed as a ratio to β-actin normalized to baseline controls, NG P0 (P0 =  before start of treatment). [*p<0.05 compared to NGP0; ‡p<0.05 compared to respective NG passages for HG cultured cells]. (B) SIRT1 enzyme activity was reduced in HG in these endothelial cells (P5, data normalized to NG). (NG = 5 mM; HG = 25 mM glucose; P = passage number). [*p<0.05 compared to NGP5].

**Figure 5:**
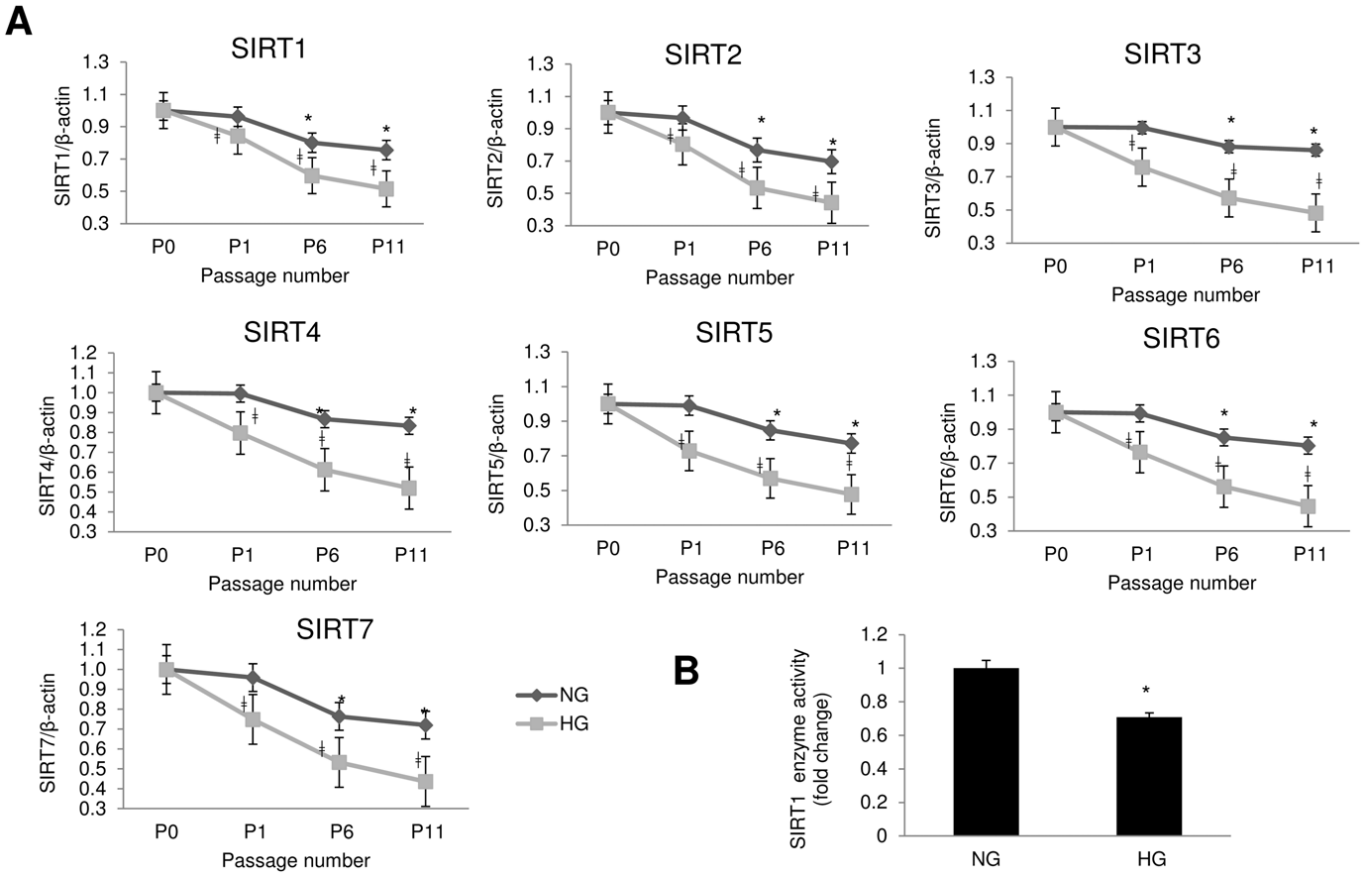
SIRT (1–7) mRNA reductions with increasing passage number is augmented with HG treatment in HUVECs. (A) SIRT (1–7) mRNA expressions are decreased in large vessel endothelial cells (HUVEC) with increasing passages and with HG it is escalated (analyzed by quantitative Real Time RT-PCR). mRNA levels are expressed as a ratio to β-actin and normalized to baseline controls, NG P0 (before treatment began). [*p<0.05 compared to NGP0; ‡p<0.05 compared to respective NG passages for HG cultured cells]. (B) SIRT1 enzyme activity was reduced in HG in HUVEC (P11, data normalized to NG). [*p<0.05 compared to NGP11]. (NG = 5 mM; HG = 25 mM glucose, P = passage number).

We then examined whether above changes are associated with alteration of SIRTs. We found mRNA expressions of all SIRT (1–7) genes were reduced with increasing passages, in all ECs (Figure 4A, Figure S2A & Figure 5A). Such reduction was faster and exaggerated in HG treated groups. There were 25–50% reductions in SIRT mRNA expressions with HG from as early as passage 1 in HMECs, which increased to 50% at passage 5 (Figure 4A). Similar reductions were observed in the BRECs (Figure S2A). HUVECs however, exhibited smaller reduction (15–30%) in earlier passages (Figure 5A). Nonetheless by passage 11, mRNA levels in HG in HUVEC were reduced to almost half of NG treated cells (Figure 5A). Reduction in SIRT1 enzyme activities found in the ECs in final passages with HG treatment, were in keeping with the reduced mRNA levels (Figure 4B, Figure S2B & Figure 5B). As SIRT1 is one of the most important isoforms of the SIRT family which is associated with aging [5], [18], [24], [34], we focused on SIRT1 for the subsequent experiments. We carried out the following experiments in HMECs, as they are the major target of diabetic complications.

### SIRT1 Activators Reduce Glucose Induced Accelerated Aging

**Figure 6:**
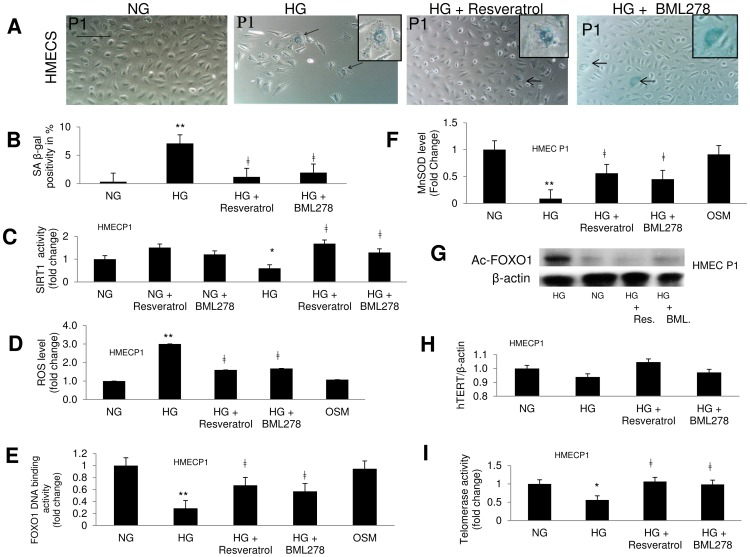
Chemically induced activation of SIRT1 reduces oxidative stress in HG treated endothelial cells. (A) SA β-gal staining of HMEC (P1) with resveratrol and BML278 treatment. β-gal positivity was reduced with SIRT1 activators in HG treated cells compared to controls. [Scale bar represent 100 µm for all micrographs]. (B) Quantification of β-gal positivity. (C) SIRT1 activators significantly increased the enzyme’s activity and reduced (D) total ROS levels in HG. (E) FOXO1 DNA binding activity was reduced in the nuclear fractions of HMEC with SIRT1 activators along with increased (F) MnSOD levels. (G) Western blot analysis of acetylated FOXO1 shows HG induced increase in Ac-FOXO1 level was corrected with SIRT1 activators. HG caused (H) nonsignificant change in TERT mRNA expression, however (I) telomerase activity was significantly reduced in the ECs and such reductions were prevented with SIRT1 activators. mRNA levels are expressed as a ratio to β-actin. All data were normalized to NG. Osmotic controls with L-glucose had no effect. [*p<0.05, **p<0.01 compared to NG; ‡p<0.05 compared to HG].

As SIRT1 enzyme is significantly reduced in HG induced oxidative stress and aging, we investigated whether SIRT1 activators can rescue such process. Both resveratrol and BML278 significantly increased SIRT1 enzyme activity (Figure 6C) and reduced the sign of aging in HG as seen from reduced β-gal positivity (Figure 6A and B). As expected, SIRT1 activators reduced total ROS levels in the HG treated cells compared to the controls (Figure 6D).

### SIRT1’s Action in ECs is Mediated through FOXO1

To elucidate the mechanism of HG induced SIRT1 mediated oxidative stress and aging pathway, we looked into FOXO1, a major regulator of MnSOD and also a target of SIRT1 [17], [23], [24]. We observed FOXO1’s DNA binding activity in nuclear fractions of HMEC was reduced in HG and effectively rescued by the SIRT1 activators (Figure 6E). In parallel, investigation of MnSOD levels in these samples correlated with the reduced FOXO1 activity levels (Figure 6F). Finally Western blot analysis demonstrated, HG induced increased acetylated FOXO1 (Ac-FOXO1) levels were efficiently reduced with SIRT1 activators (Figure 6G). Together these results supported SIRT1 regulated MnSOD pathway in the ECs during HG induced rapid aging.

We further examined TERT mRNA expressions and telomerase activity in the ECs with SIRT1 activators. In passage 1 although TERT mRNA was non-significantly reduced, telomerase activity were reduced significantly in HG and such reductions were prevented by SIRT1 activators (Figure 6H and I).

### FOXO1 Inhibitor or SIRT1 Silencing in NG Mimics the HG Effects

**Figure 7:**
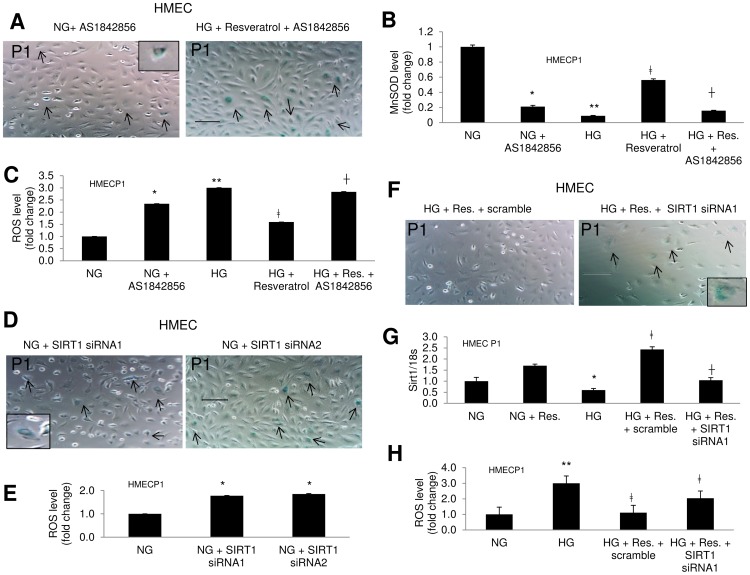
SIRT1 knockdown or FOXO1 inhibition in NG induces signs of early aging mimicking the HG treatment. (A) Early senescence was induced with FOXO1 inhibitor (AS1842856) in NG, and the rescue of senescence with resveratrol in HG disappeared with FOXO1 inhibitor, as evidenced by SA-β gal stain (HMECP1). (B) In NG, AS1842856 reduced MnSOD and increased (C) ROS levels; it also hindered the preventative effect of resveratrol in HG regarding these parameters. [*p<0.05, **p<0.01 compared to NG; ‡p<0.05 compared to HG; †p<0.05 compared to HG+Resveratrol]. (D) SIRT1 knockdown with siRNA induced senescence in NG as seen using SA-β gal stain (HMECP1). (E) Such knockdown also increased ROS levels. siRNA1 and siRNA2 represent separate experiments using two different siRNAs. (F) Glucose induced increased SA β-gal positivity was reduced with resveratrol. However SIRT1 siRNA transfection prevented such effect. (G) Analysis of SIRT1 mRNA level showed such effect of resveratrol is mediated through SIRT1. mRNA levels are expressed as a ratio to 18s normalized to controls. (H) Glucose induced increased ROS production was prevented by resveratrol. However such preventative effects were partially lost with SIRT1 siRNA transfection. [*p<0.05, **p<0.01 compared to NG; ‡p<0.05 compared to HG; †p<0.05 compared to HG+Res.+scramble]. (NG = 5 mM; HG = 25 mM glucose, P1 =  passage 1). [Scale bar represent 100 µm for all micrographs].

To investigate the role of FOXO1 in such pathway further, we treated HMECs with a commercially available FOXO1 inhibitor (AS1842856) in NG. As expected FOXO1 inhibitor reduced FOXO1 DNA binding (data not presented) and subsequently reduced the MnSOD levels (Figure 7B). This led to an increase in ROS levels and induced early senescence as seen from increased β-gal positivity (Figure 7A and C). In addition, FOXO1 inhibitor dampened the preventative effects of resveratrol on glucose-induced aging and oxidative stress, supporting the relationship of SIRT1 and FOXO1 (Figure 7A–C). Silencing of SIRT1 expression with siRNA (>50% reduction in mRNA expression, data not shown) in NG also showed similar effects and mimicked the HG effects (Figure 7D and E). These findings confirmed our earlier findings and the specificity of SIRT1 and FOXO1 in the rapid aging pathway in these ECs.

We then performed some additional experiments using SIRT1 siRNA in ECs following SIRT1 activation with resveratrol. We treated HMECs with SIRT1 siRNA for 24 hrs following treatment with resveratrol for 48 hrs. We found the siRNA reversed the effect of resveratrol on mRNA expression, oxidative stress and aging (Figure 7F–H). This re-demonstrated the SIRT1 mediated aging in the ECs in hyperglycemia.

### SIRT1 and p300 have a Balancing Role on Each Other

**Figure 8:**
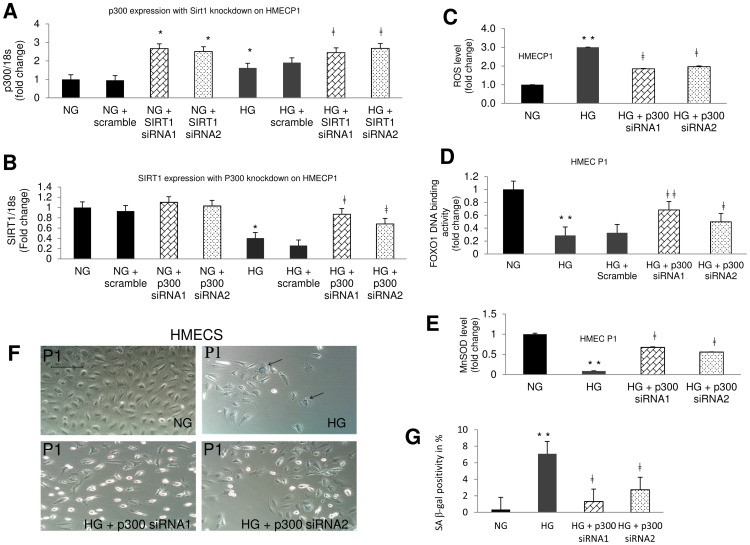
SIRT1 and p300 regulate each other in microvascular endothelial cells. (A) Quantitative Real Time RT-PCR analysis showed HG upregulated p300 mRNA and such effects are augmented by SIRT1 knockdown with siRNA. (B) HG induced downregulation of SIRT1 mRNA expression was corrected with p300 knockdown with siRNA. mRNA levels are expressed as ratio of 18s normalized to NG. (C) p300 siRNA reduced total ROS levels in HG by increasing (D) FOXO1’s DNA binding activity subsequently increasing (E) the MnSOD levels. (F, G) Microscopic photographs and quantitative analysis showed glucose induced increased SA β-gal positivity is effectively prevented by p300 siRNA. Data normalized to NG. (NG = 5 mM; HG = 25 mM D-glucose, OSM = 25 mM L-glucose, P1 =  passage1). [*p<0.05 and **p<0.01 compared to NG; ‡P<0.05 and ‡‡p<0.01 compared to HG. Scale bar represent 100 µm for all micrographs. siRNA1 and siRNA2 represent separate experiments using two different siRNAs].

In order to explore the relationship of SIRT1 with other molecules in HG induced accelerated aging, we investigated possible association between SIRT1 and p300. Since SIRT1 is a HDAC and actions of deacetylases are balanced by HATs, we examined p300, a well characterized HAT. To investigate such phenomenon, we knocked down SIRT1 in HMECs using siRNA and examined p300 mRNA expression and vice versa (Figure 8A and B). We were able to achieve more than 50% reduction of the specific mRNAs following the transfections (data not shown). SIRT1 silencing increased p300 mRNA expression in both NG and HG treated cells (Figure 8A). On the other hand, p300 silencing had no significant effect on basal SIRT1 mRNA level but reversed glucose induced reduction of SIRT1 expression indicating a possible regulatory role on each other (Figure 8B). To explore this relationship further we performed additional experiments investigating the downstream molecules (Figure 8C–F). We found p300 silencing in HG lead to a significant increase in FOXO1 DNA binding activity and subsequent MnSOD level compared to controls (Figure 8C and D). In addition, glucose induced increase of ROS and β-gal positivity were also reduced with p300 siRNA (Figure 8E–G). These findings together indicate that both SIRT1 and p300 regulate oxidative stress pathway in ECs through FOXO1.

### Diabetes Causes Accelerated Aging in Kidney

**Figure 9:**
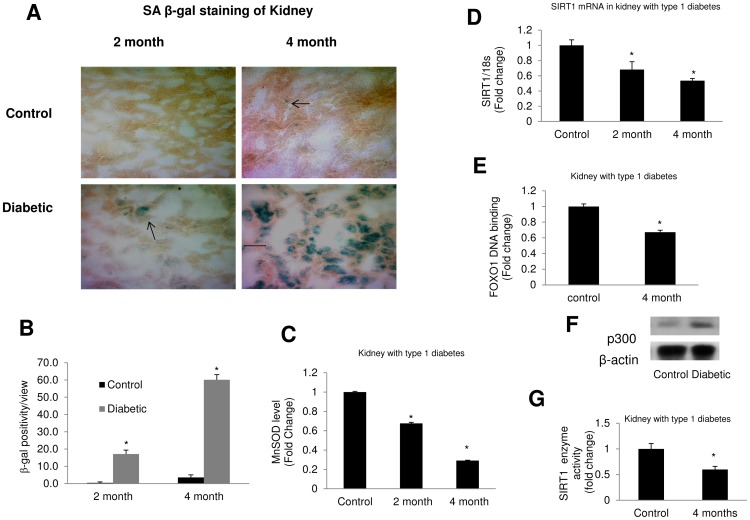
Diabetes causes accelerated aging in mice kidney. (A) SA β-gal staining of kidney tissue of STZ induced diabetic mice at 2 and 4 months showing increased positivity with uncontrolled diabetes. Arrow indicates β-gal positive cells. (B) Quantification of β-gal staining of the kidney tissue. Data presented as β-gal positivity/view (n = 10 image). (C) Shows reduction of MnSOD levels in kidney tissues of the diabetic animals following 2 and 4 months. (D) mRNA analysis confirmed downregulation of SIRT1 expressions in the kidney tissues of these animals. mRNA levels are expressed as a ratio to 18 s normalized to controls. (E) FOXO1 DNA binding and (G) SIRT1 enzyme activities are reduced in the kidney tissues in diabetes. (F) Western blot analysis showed increased p300 protein levels in the kidneys in diabetes. Data normalized to controls. [*p<0.05 compared to control animals. Scale bar represents 100 µm for all micrographs].

Finally, in order to investigate if the findings *in vitro* is reflected *in vivo*, we used a well-established animal model, STZ induced C57BL/6 mice. Diabetic animals showed high blood glucose (23.43±3.27 mmol/L vs. controls 7.3±0.93 mmol/L, *p*<0.001) and reduced body weight (22.50±1.11 gm vs. controls 30.25±2.04 gm, *p*<0.003). Urinary albumin concentration was increased (0.58±0.07 µg/mL vs. 0.21±0.07 µg/mL in control, *p*<0.009) along with increased serum creatinine in the diabetic animals (0.52±0.08 mg/dL vs. controls 0.18±0.04 mg/dL, *p*<0.02). Kidney tissues from diabetic mice showed increased β-gal positivity compared to the controls, supporting our *in vitro* findings (Figure 9A and B). This was further demonstrated as MnSOD levels, FOXO1 transcriptional activity, SIRT1 mRNA expression and enzyme activity were significantly reduced in kidneys of the diabetic animals along with an increased p300 protein levels in these tissues (Figure 9C–G).

**Figure 10:**
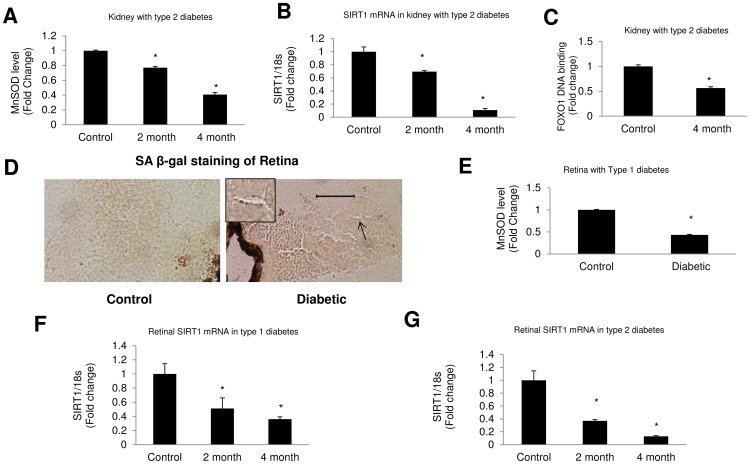
Oxidative stress and associated changes are present in the kidneys and retinas of diabetic animals. (A) MnSOD levels were reduced in kidneys of *db/db* mice following 2 and 4 months of diabetes indicating increased oxidative stress. (B, C) SIRT1 mRNA expression and FOXO1 activity in kidneys of *db/db* mice were reduced compared to controls. (D) SA β-gal staining of type 1 diabetic mice retina showed increased positivity in retinal blood vessels following 2 months of diabetes. [Scale bar represent 50 µm for all micrographs]. Arrow indicates retinal blood vessels. (E) Such changes were associated with reduced MnSOD level in these tissues. (F, G) Shows reduction of SIRT1 mRNA expressions in retinal tissues of both type 1 and 2 diabetic mice. mRNA levels are expressed as a ratio to 18s normalized to controls. [*p<0.05 compared to control animals].

We further examined renal tissues from type 2 diabetic mice that were hyperglycemic and obese (data not shown). We found reduced MnSOD level following 2 and 4 months of diabetes in the kidney tissues of these animals (Figure 10A). mRNA analysis further showed reduced SIRT1 expression in these tissues (Figure 10B). In addition FOXO1 DNA binding activity was reduced in the kidneys of these mice (Figure 10C).

### SIRT1 Expression is Reduced in Retina with Type 1 and Type 2 Diabetes

Furthermore, we examined SIRT1 mRNA expression in retinas of the type 1 and 2 diabetic animals. Retinal SIRT1 mRNA expressions were reduced in both models following 2 months of diabetes and the effects were pronounced after 4 months (Figure 10F and G). Further, SA β-gal staining of mice retina with type 1 diabetes showed increased positivity in the blood vessels following 2 months of diabetes, along with reduced antioxidant levels in these tissues (Figure 10D and E).

## Discussion

This study demonstrates that hyperglycemia accelerates aging both *in vitro* and *in vivo*. Although some studies have previously been performed [35] on ECs investigating the effects of HG exposure for short term periods (24 to 72 hr), no study has yet been reported on chronic HG exposure of these cells, to simulate the process of chronic hyperglycemia in an *in vitro* setting. We simulated chronic hyperglycemia in the ECs with HG and propagated them in HG for weeks or even months until the cells stopped proliferation completely for more than 2 weeks. Such design allowed us to examine glucose induced accelerated aging process amongst ECs of different origins. We have seen signs of cellular senescence by SA β-gal positivity early in microvascular ECs and retinal ECs. However this process was slower in large vessel ECs. The differentiated ECs were more susceptible to hyperglycemia induced damage compared to HUVEC, which is more close to progenitor cells in terms of differentiation [36] and are a better survivor in hyperglycaemia. We have demonstrated that such process was associated with increased oxidative stress.

Molecular diversity in the genetic level in ECs of various origins has been studied before [37], [38]. It has been shown that the gene expression profiles of ECs from large vessels are different from those of micro-vessels [39]. In keeping with these findings our study further demonstrated and elaborated some structural and functional significance of these type of changes.

We have shown an important role played by SIRTs in this study. Since the discovery of SIRTs, interests in these deacetylases have generated multiple lines of evidences indicating SIRTs as evolutionarily conserved regulators of lifespan [9], [18]–[21], [40]. SIRTs regulate physiological response to metabolism and stress, the two key factors affecting the aging process. We found all SIRT1–7 mRNA expressions were consistently reduced with the accelerated aging process in hyperglycemia. Although isolated SIRT alterations have been demonstrated in various studies [19], [20], [41]–[43], such comprehensive analysis of SIRTs have not been done. More interestingly, the level of SIRTs reduction paralleled that of β-gal positivity and such changes were prevented by SIRT activators indicating an important relationship between SIRTs and aging.

SIRT1, the leading enzyme in the SIRT family is found both in the cytoplasm and nucleus and has been found to have protective roles in stress resistance and cell survival in various diseases [41], [44], [45]. This study showed glucose induced alteration of downstream mediators of SIRT1, such as FOXO1 were corrected by SIRT1 activators further establishing the crucial role of SIRT1 in this process. The regulatory role of SIRT1 and p300 was further interesting. p300 and SIRT1 regulates each other as silencing one gene lead to increased expression of the other gene producing downstream affects. Other studies have shown, in a different model, p300 acetylates and activates PPAR-γ leading to its binding to SIRT1 promoter, thus decreasing SIRT1 expression [44]. Hence both direct and indirect relationship between these two molecules may exist in the ECs.

We have previously shown that p300 regulates multiple transcription factors and proteins [6], [16]. Other studies have also shown acetylation of FOXO1 by p300 facilitates its phosphorylation by Akt ultimately exporting it from the nucleus with chaperon 14-3-3 protein [24], [46]. In line with that, this study further showed HG induced upregulation of p300 leads to attenuation of FOXO1 DNA binding activity in the ECs. This led to a reduction in MnSOD level, a known target gene of FOXO1 [27] and increased oxidative stress in the ECs. Furthermore, silencing p300 with siRNA corrected the MnSOD reduction and downstream changes in hyperglycemia, confirming such pathway.

On the other hand as FOXO1 deacetylation by SIRT1 is necessary for its retention in the nucleus [10], [29], we also found increased FOXO1 DNA binding with SIRT1 activators, which increased SIRT1 enzyme activity and reduced SA β-gal positivity. Additionally, both FOXO1 inhibitor and SIRT1 siRNA was able to induce early senescence in NG, and mimicked the HG effect in the ECs. Together, these results indicate an important mechanistic pathway of oxidative stress and aging in HG, mediated through FOXO1 and regulated by SIRT1 and p300.

In keeping with the *in vitro* studies, STZ induced diabetic mice showed increased β-gal positivity in the kidneys following 2 & 4 months of uncontrolled diabetes. The retina showed increased signs of aging following 2 months of diabetes. This was accompanied by significantly reduced antioxidant levels in the tissues. In addition, SIRT1 mRNA was found to be significantly reduced in kidney and retina of both type 1 and type 2 diabetic mice.

In summary, this study showed chronic hyperglycemia accelerated aging process through a novel SIRT1 and p300 regulated pathway. We demonstrated HG induced reduction in SIRT1 lead to increased oxidative stress mediated through FOXO1 which was prevented by SIRT1 activation. We further demonstrated p300 negatively regulates such pathway, as silencing p300 mimicked resveratrol’s effect. Identification of such novel mechanisms will allow us to better understand the pathogenesis of diabetic complications as well as aging and eventually help to find potential therapeutic treatment.

## Supporting Information

Figure S1 **Accelerated aging and associated changes in retinal endothelial cells (BREC) with increasing passages with HG treatment.** (A) SA-βgal staining showing increased β-gal positivity (A) with HG treatment. Arrow indicates β-gal positive cells. [Scale bar represent 100 µm for all micrographs]. (B) Quantification of β-gal positivity. Aging signs appeared at passage 1 with HG. (C) Morphometric cell area analysis showed significant increase in cell size with HG treatment. Cell growth was slow with HG treatment compared to controls as seen from (D) days needed to be confluent (90%, at which stage cells were subcultured to next passage), (E) cell count (attached cells per microscopic field at 20× objective, n = 10 image) and (F) reduced intracellular LDH activity. (NG = normal glucose, 5 mM; HG = high glucose, 25 mM D-glucose; OSM = osmotic control, 25 mM L-glucose; P = passage number). [*p<0.05, **p<0.01 compared to NGP1; ‡p<0.05, ‡‡p<0.01 compared to respective NG passages for HG cultured cells].
(TIF)

Figure S2 **SIRT 1–7 mRNA analysis with Quantitative Real Time RTPCR at various passages in BREC showed significant reduction in HG treated cells.** (A) SIRT (1–7) mRNA expressions in BREC with increasing passages. mRNA levels are expressed as a ratio to β-actin and normalized to baseline controls, NG P0 (before treatment began). [*p<0.05 compared to NGP0; ‡p<0.05 compared to respective NG passage for HG cultured cells]. (B) SIRT1 enzyme activity was reduced in HG in BREC (P8, data normalized to NG). (NG = 5 mM; HG = 25 mM glucose; P = passage number). [*p<0.05 compared to NGP8].
(TIF)

Table S1 **The primers sequences for Real Time RT-PCR.**
(DOCX)
