# Supplementary figures and images for: High Glucose Induced Alteration of SIRTs in Endothelial Cells Causes Rapid Aging in a p300 and FOXO Regulated Pathway
Source: PLoS One. 2013 Jan 16;8(1):e54514. doi: 10.1371/journal.pone.0054514 (PMC3546959; doi:10.1371/journal.pone.0054514)

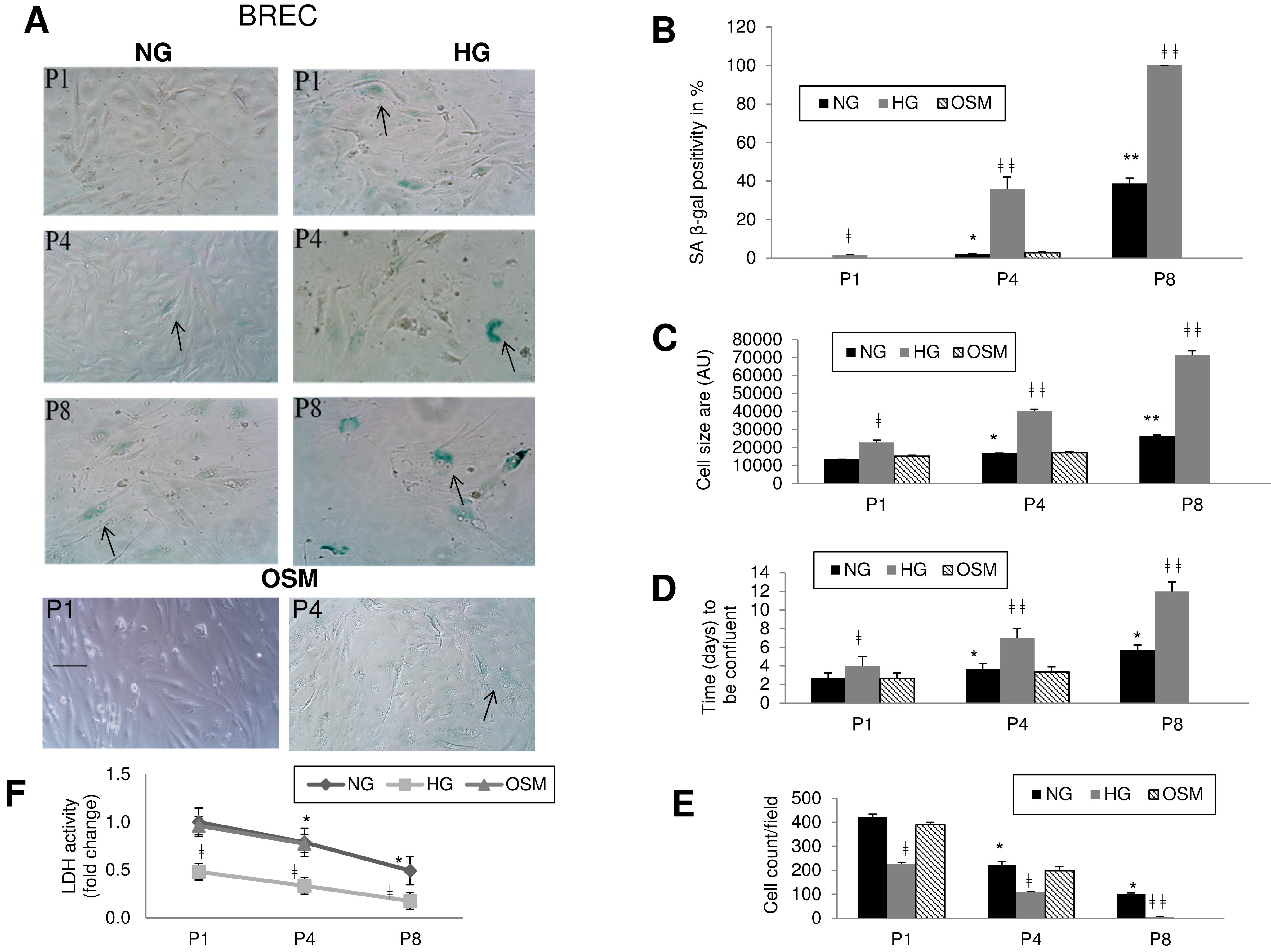

Supplement: Figure S1 — Accelerated aging and associated changes in retinal endothelial cells (BREC) with increasing passages with HG treatment. (A) SA-βgal staining showing increased β-gal positivity (A) with HG treatment. Arrow indicates β-gal positive cells. [Scale bar represent 100 µm for all micrographs]. (B) Quantification of β-gal positivity. Aging signs appeared at passage 1 with HG. (C) Morphometric cell area analysis showed significant increase in cell size with HG treatment. Cell growth was slow with HG treatment compared to controls as seen from (D) days needed to be confluent (90%, at which stage cells were subcultured to next passage), (E) cell count (attached cells per microscopic field at 20× objective, n = 10 image) and (F) reduced intracellular LDH activity. (NG = normal glucose, 5 mM; HG = high glucose, 25 mM D-glucose; OSM = osmotic control, 25 mM L-glucose; P = passage number). [*p<0.05, **p<0.01 compared to NGP1; ‡p<0.05, ‡‡p<0.01 compared to respective NG passages for HG cultured cells]. (TIF) [file pone.0054514.s001.tif]

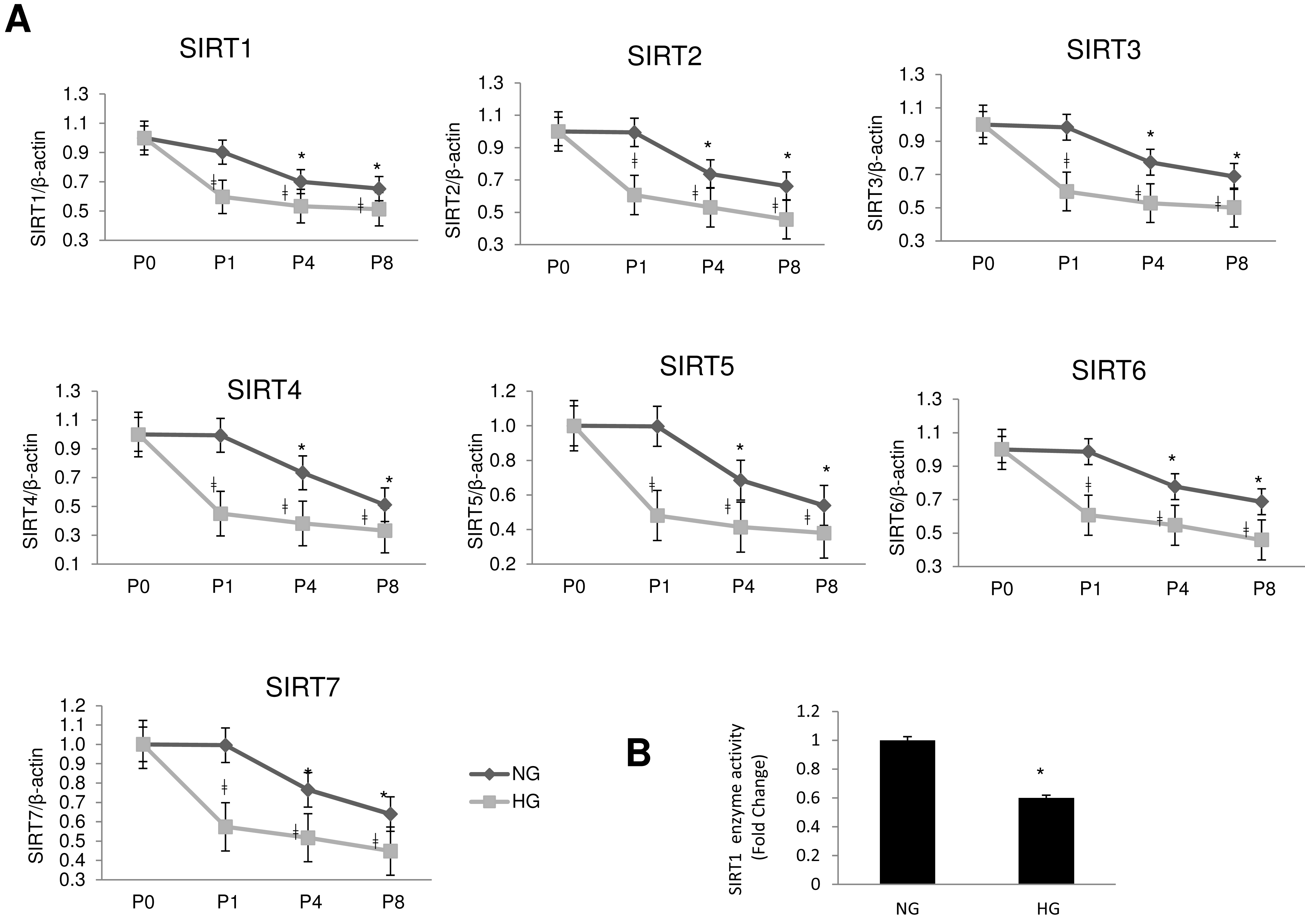

Supplement: Figure S2 — SIRT 1–7 mRNA analysis with Quantitative Real Time RTPCR at various passages in BREC showed significant reduction in HG treated cells. (A) SIRT (1–7) mRNA expressions in BREC with increasing passages. mRNA levels are expressed as a ratio to β-actin and normalized to baseline controls, NG P0 (before treatment began). [*p<0.05 compared to NGP0; ‡p<0.05 compared to respective NG passage for HG cultured cells]. (B) SIRT1 enzyme activity was reduced in HG in BREC (P8, data normalized to NG). (NG = 5 mM; HG = 25 mM glucose; P = passage number). [*p<0.05 compared to NGP8]. (TIF) [file pone.0054514.s002.tif]
