# Supplementary material for: High Glucose Induced Alteration of SIRTs in Endothelial Cells Causes Rapid Aging in a p300 and FOXO Regulated Pathway
Source: PLoS One. 2013 Jan 16;8(1):e54514. doi: 10.1371/journal.pone.0054514 (PMC3546959; doi:10.1371/journal.pone.0054514)
Supplement: Table S1 — The primers sequences for Real Time RT-PCR. (DOCX) [file pone.0054514.s003.docx]

| **Name of Primer** | **Sequence (5’ to 3’)** |
| --- | --- |
| TERT | CGTGGTTTCTGTGTGGTGTC  CCTTGTCGCCTGAGGAGTAG |
| FOXO1 | AAGAGCGTGCCCTACTTCAA  CTGTTGTTGTCCATGGATGC |
| SIRT3 | CATGAGCTGCAGTGACTGGT  GAGCTTGCCGTTCAACTAGG |
| SIRT4 | CAGCAAGTCCTCCTCTGGAC  CCAGCCTACGAAGTTTCTCG |
| SIRT6 | AGGATGTCGGTGAATTACGC  AAAGGTGGTGTCGAACTTGG |
| SIRT2 | GACTTTCGCTCTCCATCCAC  GGAGTAGCCCCTTGTCCTTC |
| SIRT5 | CCAGATTGTCCCAAGTCGAT  CTGAAGGTCGGAACACCACT |
| SIRT7 | AGGGAGAAGCGTTAGTGCTG  GGAACGCAGGAGGTACAGAC |
| SIRT1 | GCAGATTAGTAGGCGGCTTG  TCTGGCATGTCCCACTATCA |
| P300 | GGGACTAACCAATGGTGGTG  ATTGGGAGAAGTCAAGCCTG |
